# Correlation between Autoimmune Hashimoto’s Thyroiditis and *Helicobacter pylori* Infection: A Case-Control Study

**DOI:** 10.34172/mejdd.2024.397

**Published:** 2024-10-30

**Authors:** Mahla Shajari, Maryam Rezaei, Fereshteh Osmani, Ebrahim Shafaie, Zoya Tahergorabi

**Affiliations:** ^1^Student Research Committee, School of Medicine, Birjand University of Medical Sciences, Birjand, Iran; ^2^Department of Internal Medicine, School of Medicine, Medical Toxicology and Drug Abuse Research Center, Birjand University of Medical Sciences, Birjand, Iran; ^3^Department of Epidemiology and Biostatistics, School of Health, Infectious Diseases Research Center, Birjand University of Medical Sciences, Birjand, Iran; ^4^School of Medicine, Infectious Diseases Research Center, Birjand University of Medical Sciences, Birjand, Iran; ^5^Geriatric Health Research Center, Department of Physiology, School of Medicine, Birjand University of Medical Sciences, Birjand, Iran

**Keywords:** Helicobacter pylori, Hashimoto’s thyroiditis, Thyroid peroxidase antibody, Thyroid stimulating hormone

## Abstract

**Background:**

Among environmental factors, infectious agents, including *Helicobacter pylori*, can act as triggers for autoimmune thyroid diseases. Therefore, this study aimed to investigate the correlation between autoimmune Hashimoto’s thyroiditis with *H. pylori* infection.

**Methods:**

The participants in this case-control study were 74 individuals 17-62 years who were divided into two groups, including 38 diagnosed Hashimoto’s thyroiditis patients from an outpatient clinic of endocrinology and 36 apparently healthy individuals that were selected from family members of cases group age-matched and sex-matched. For individuals in two groups, a questionnaire was completed, including demographic information. Then, they were referred to the laboratory for thyroid stimulating hormone (TSH) and free T4 (FT4) in the control group and anti-thyroid peroxidase antibody (TPO-Ab) levels measurement in case and control groups. Stool samples were obtained from all individuals for *H. pylori* antigen detection using the ELIZA kit.

**Results:**

There was no significant difference in the mean age of case and control groups (*P*=0.96), and 81.1% of individuals were female. 58.6% of patients with Hashimoto’s thyroiditis and 41.4% of the control group had positive *H. pylori*, but there was no statistically significant difference between the two groups (*P*=0.34). Furthermore, there was a significant positive correlation between TPO-Ab levels and *H. pylori* infection (*r*=0.2, *P*=0.03).

**Conclusion:**

TPO-Ab levels were associated with *H. pylori* infection diagnosed by *H. pylori* antigen.

## Introduction

 The presence of thyroid autoantibodies in patients with atrophic gastritis proposed the thyrogastric syndrome in the early 1960s.^1^ The thyroid gland and stomach, due to their common embryonic origin, have similar morphological and functional characteristics, including the ability to concentrate and transport iodine across the gastric mucosal and thyroid follicular cell membrane, despite different localization.^1,2^

 Autoimmune thyroid diseases, as the most common autoimmune diseases, usually result from the loss of immune tolerance to autoantigens, and they include a group of autoimmune diseases such as Graves’ disease, Hashimoto’s thyroiditis, atrophic thyroiditis, and postpartum thyroiditis, etc.^3^ Hashimoto’s thyroiditis has a prevalence of approximately 5% in the general population, and also, it is highly prevalent in female sex.^4^

 Although the etiology of autoimmune thyroid diseases is multifactorial however, mounting evidence shows synergism of genetic (80%) and environmental factors (20%), such as drugs, stress, radiation, and various microorganisms play a role in promoting thyroid autoimmunity.^5^ Among microorganisms, *Helicobacter pylori* a gram-negative bacterium which is mainly found in mucus membranes of the stomach colonized in nearly half of the world population therefore it is considered the most common bacterial infection in humans.^6^ Microorganisms, including *H. pylori,* through homologous antigen-specific signals, mediate molecular stimulation or mobilization of endogenous antigens and cause inflammation, enhance the immune response, and induce autoimmunity.^7^

*Helicobacter pylori* infection is acquired in childhood and is generally transmitted from mouth to mouth and feces to mouth. Poor living conditions and low socioeconomic status in childhood play an important role in the high prevalence of disease.^8^ Proportionally, the prevalence rate of *H. pylori* infection is different in developing and developed countries (70% vs. 25%-50% of people), respectively.^9^

 Some digestive diseases have been linked with *H. pylori* infection, such as chronic gastritis, peptic ulcer disease, gastric cancer, and some extra-digestive manifestations such as thyroid mucosal associated lymphocyte tissue (MALT) lymphoma, diabetes mellitus, iron deficiency anemia, idiopathic thrombocytopenic purpura and deficiency of vitamin B12.^10^

 In this context, the coexistence of some diseases (such as celiac disease, lactose intolerance, and inflammatory bowel diseases) may interfere with treatment in patients of hypothyroidism; therefore, it is considered one of the main causes of poor control and increased need for thyroid hormone in these patients. It is especially there in patients with atrophic gastritis associated with *H. pylori* infection or individuals with impaired acid secretion in the stomach (severe hypochlorhydria, achlorhydria).^11^ Therefore, this study aimed to investigate the correlation between autoimmune Hashimoto’s thyroiditis with* H. pylori* infection.

## Methods and Materials

 In this case-control study, 38 samples from patients with Hashimoto’s thyroiditiswere diagnosed by the endocrinologist according to high thyroid peroxidase antibody (TPO-Ab) levels as case group (because they were diagnosed patients therefore only was checked TPO-Ab) and 36 samples from individuals with normal thyroid function obtained as control group according to negative TPO, TSH, and free T4 (FT4) levels according to American Thyroid Association recommendation for diagnosis both outpatient and hospitalized patients.^12^ The patients were selected from an outpatient clinic of endocrinology in Birjand (capital city in South Khorasan province) and the control group who were asymptomatic from the perspective of Hashimoto’s thyroiditis selected from family members of cases group and were matched based on age, sex, and social and economic status.

 Using the results obtained from the study of Mohammed Nabil Raafat et al,^3^ according to the following formula and considering the power of 90% and the confidence level of 90%, the sample size of 36 cases in each group was determined.


$$n=\frac{{\left(Z_{1-\alpha /2}+Z_{1-\beta }\right)}^{2}\left(\sigma_{1}^{2}+\sigma_{2}^{2}\right)}{d^{2}}$$

Inclusion criteria were patients with at least 1 year diagnosis of Hashimoto’s thyroiditis, absence of other diseases, and negative antimicrobial drug use for the past 3 months.Exclusion criteria were patients with any type of thyroid disorder, autoimmune diseases, gastrointestinal diseases, and antibiotic use in 3 recent months.

 A questionnaire consisting of demographic information and history of previous gastrointestinal diseases in participants and their families was completed for case and control groups. Then, individuals in the case and control groups were referred to a laboratory to take 5 mL venous blood samples for TPO antibody levels or TSH, FT4, and TPO antibody levels measurement in serum, respectively using kit (LIAISON, DiaSorin S.p.A, Italy) and TPO antibody levels measurement in serum with the electrochemiluminescence immunoassay system (LIAISON, DiaSorin, Germany). Also, stool samples were kept frozen at -70 ^º^C for *H. pylori *antigendetection by ELISA kit. The procedure was applied according to the manufacturer’s instructions (ELISA kit PISHTAZ TEB, Tehran, Iran with sensitivity = 99.04% and specificity = 99.29% and Biotech ELISA reader and washer (Biotech, USA). TSH, TPO antibody, and FT4 values more than 6.3 MIU/L, 16 iu/mL and 2.2 ng/mL were considered positive, respectively.

 The SPSS software was used for statistical analyses (SPSS 19, Chicago, IL, USA). The Kolmogorov-Smirnov test was used to detect the normality of variables. The results are provided using descriptive statistics, including frequency, percentage, and mean with standard deviation (SD). Mann-Whitney test was applied to compare the median difference of numerical variables between study groups. *Pearson’s chi-square test* or Fisher’s exact test was used to investigate the association between categorical variables among study groups. TPO-Ab with *H. pylori* antigen levels was assessed using the Pearson’s correlation coefficient test. Statistical significance was considered when the *P* < 0.05.

## Results

**Figure 1:**
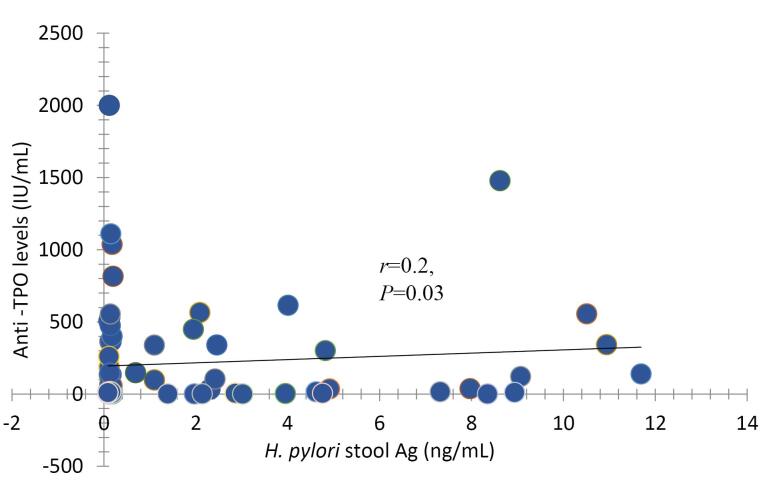
Pearson's correlation between TPO-Ab levels and *Helicobacter pylori* infection

 In our study, there were 74 individuals of 17-62 years, including 38 patients with Hashimoto’s thyroiditisas a case group and 36 healthy individuals as a control group. Table 1 shows the demographic characteristics of individuals in two groups. With respect to age, the mean age ± SD of case and control groups were identical (36.55 ± 8.83 vs. 36.66 ± 12.03, *P* = 0.96), respectively. 86.8% of patients and 75% of the control group were female, andinregard to sex both groups were identical (*P* = 0.24).Dyspepsia and gastroesophageal reflux disease (GERD) were the most common gastrointestinal symptoms in the case group compared with the control group (*P* = 0.003**),** and mother and sister had the most history of thyroid diseases among relatives of patients compared with the control group (*P* = 0.003**)**. The mean TPO-Ab levels in the case group were significantly higher than the control group (411.39 ± 424.82 vs.5.96 ± 5.87, *P* < 0.001) iu/mL, respectively (Table 2). Also, our results showed that 17 (58.6%) patients with Hashimoto’s thyroiditiscompared with 12 (41.4%) of the control group had positive* H. pylori *stool antigen; however, there was no significant difference between the two groups (*P* = 0.34, Table 3). In addition, there was a significant positive correlation between TPO-Ab levels and *H. pylori* infection (r = 0.2, *P* = 0.03, Figure 1).

**Table 1:** Demographic characteristics of the case and control groups

**Parameter**	**Subgroup**	**Control group (n=36) [n (%)]**	**Hashimoto’s thyroiditis group (n=38) [n (%)]**	**Total (n=74) [n (%)]**	***P*** ** value (Chi-square test)**
Sex	Male	9 (25.0%)	5 (13.2%)	14 (18.9%)	0.240
	Female	27 (75.0%)	33 (86.8%)	60 (81.1%)	
Marriage	Married	27 (75.0%)	36 (94.7%)	63 (85.1%)	0.017*
	Single	9 (25.0%)	2 (5.3%)	11 (14.9%)	
Residence	urban	36 (100%)	34 (89.5%)	70 (94.6%)	0.064
	rural	0 (0.0%)	4 (10.5%)	4 (5.4%)	
Occupation	Non-government employee	12 (33.3%)	9 (23.7)	21 (28.4%)	0.136
	Government employee	7 (19.4%)	7 (18.4%)	14 (18.9%)	
	Retired	2 (5.6%)	0 (0.0%)	2 (2.7%)	
	Housekeeper	10 (27.8%)	20 (52.6%)	30 (40.5%)	
	Student	5 (13.9%)	2 (5.3%)	7 (9.5%)	
Income	1-3 million	3 (8.3%)	5 (13.2%)	8 (10.8%)	0.268
	> 3 million	20 (55.6%)	14 (36.8%)	34 (45.9%)	
	No income	13 (36.1%)	19 (50.0%)	32 (43.2%)	
Education	under diploma	3 (8.3%)	8 (21%)	11 (14.9%)	0.087
	Diploma	16 (44.4%)	14 (36.8%)	30 (40.5%)	
	University	17 (47.2)	16 (42.1%)	33 (44.6%)	
History of thyroid diseases in relatives	Mother and sister	2 (5.6%)	15 (39.5%)	17 (23.0%)	0.003*
	Others (daughter, aunt, uncle, niece and nephew, spouse, mother-in-law)	16 (44.4%)	9 (23.6%)	25 (33.8%)	
	No	18 (50%)	14 (36.8%)	32 (43.2%)	
Having gastrointestinal symptoms	Yes	0 (0.0%)	23 (60.5%)	23 (3.1%)	< 0.001**
	No	36 (100%)	15 (39.5%)	51 (68.9%)	
Gastrointestinal symptoms	Gastric pain	0 (0.0%)	2 (5.3%)	2 (2.7%)	0.003*
	Dyspepsia	0 (0.0%)	5 (13.2%)	1 (1.4%)	
	Gastroesophageal reflux disease	0 (0.0%)	4 (10.5%)	4 (5.4%)	
	Heartburn	0 (0.0%)	2 (5.3%)	2 (2.7%)	
	Constipation	0 (0.0%)	2 (5.3%)	2 (2.7%)	
	Gastric pain & reflux	0 (0.0%)	2 (5.3%)	2 (2.7%)	
	Dyspepsia& reflux	0 (0.0%)	2 (5.3%)	2 (2.7%)	
	Others (heaviness in stomach)	0 (0.0%)	4 (10.5%)	4 (5.4%)	
	Without symptom	36 (100%)	15 (39.5%)	51 (68.9%)	

* *P* value less than 0.05. **P value less than 0.001.

**Table 2:** The comparison of anti-TPO levels between the case and control groups

**Parameter**	**Hashimoto’s thyroiditis group (n=38) (Mean±SD)**	**Median (Q1-Q3)**	**Control group (n=36) Mean±SD]**	**Median (Q1-Q3)**	**Total (n=74) (Mean±SD)**	***P*** **value** ^a^
Anti-TPO (IU/mL)	411.39 ± 424.89	339.50 (117.50-555.25)	5.96 ± 5.87	3.50 (0.90-10.75)	214.15 ± 364.85	< 0.001**

Anti-TPO: anti-thyroid peroxidase. ^a^ Mann–Whitney U test. **P value less than 0.001 when compared with the control group.

**Table 3:** Frequency of *Heliobacter pylori* in the case and control groups

**Group**	***Helicobacter pylori***		***P*** ** value**
	**Positive, No. (%)**	**Negative, No. (%)**	
Hashimoto’s thyroiditis	17 (58.6%)	21 (46.7%)	0.34
Control	12 (41.4%)	24 (53.3%)	

^a^ Chi-square test.

## Discussion

 A number of studies have postulated that among microorganisms, *H. pylori* can play a significant pathogenic role in autoimmune disorders, including Hashimoto’s thyroiditis; however, the results are controversial.^13,14^ Different techniques used to assess *H. pylori* infection may able to explain these conflicting results. Also,Hashimoto’s thyroiditiswith different grades of thyroid function, such as subclinical or frank hypothyroidism, can be another factor responsible for conflicting conclusions.^15^

 Our results showed that the age range was 17-62 years, which is in agreement with the study by Abo El Azaim et al.^16^ Emerging evidence shows that thyroid dysfunction mostly occurs in adult age and its prevalence increases with age but may be seen in any age group including children.^17^ In our study, there was no significant difference between the two groups in terms of age to avoid the influence of age as a confounding factor.

 Furthermore, 86.8% of participants in the case group were female, which is in agreement with Hamid’s study, where 82% of Hashimoto’s thyroiditis patients were females.^18^ A possible explanation is that there is a direct interaction between immune system cells and sex hormones via the receptor or inside immune cells therefore, sex hormones like estrogen, progesterone, and testosterone can play roles in many immune responses. Therefore, there are sex-based differences in the prevalence of autoimmune disease.^19^

 Hashimoto’s thyroiditisin our study was considered based on clinical criteria: increased TSH, low free T4, positive anti-TPO, and measurement of TPO antibody used in our study as a strong parameter in the screening for thyroid autoimmunity. Anti-TPO and anti-Tg are major anti-thyroid antibodies that are widely used in clinical laboratories to diagnose Hashimoto’s thyroiditis disease.Siriwardhane et al showed that measurement of these antibodies was beneficial for early prediction of the development of thyroid autoimmunity and recommend adding them to the list of thyroid function tests, including FT3, FT4, and TSH.^20^

 Our study indicated that 58.6% of patients in the case group had positive *H. pylori *antigen, which is in line with Hamid’s study, where 57% of patients with Hashimoto’s thyroiditisin Baghdad city had positiveanti*–HP* IgG Abs. ^18^ Possible explanation is exposure to a microbe leads to more than one autoimmune disease although its exact mechanism is not well defined but cross-reaction responses against a microbial mimic have been demonstrated.^21^

 In addition, a positive correlation between *H. pylori *infection and TPO antibody was found, which is consistent with the Rashad and Gomaa’s study on 300 individuals, including 187 individuals with normal thyroid function and 113 patients with thyroid dysfunction.^22^ Also, it is consistent with the study by Korani et al in 2016 on 40 female patients with Hashimoto’s thyroiditisas a case groupand 30 healthy females as a control group.^23^ Our result contrasts Tomasi et al study on 302 patients with signs and symptoms of dyspepsia who underwent an upper endoscopy that indicated no differences in anti-TPO and anti-thyroglobulin in patients with and without *H. pylori *infection.^14^ Possible explanation is that both thyroid and stomach have fetal and structural similarities (such as the ability to concentrate iodine, the presence of apical microvilli, synthesis and excretion of glycol protein (thyroglobulin and mucin) also, superficial parietal cell antigens of the stomach are homologous with protein portion of thyroid peroxides enzyme) may explain their simultaneous involvement of them in some diseases and *H. *pyloriinfection could trigger autoimmune reaction in thyroid gland.^24^ Also, *H. pylori *and thyroidhave similar epitopes;therefore, damage of gastric cells by *H. pylori *infection with exposure of epitopes with the immune system triggers an autoimmune response due to molecular mimicry.^25^

 In addition, in our study, dyspepsia and GERD were the most common gastrointestinal symptoms in the case group compared with the control group, which is in line with the Krakowska-Stasiak et al study on 24 patients with GERD and thyroid disease and 36 patients with GERD without an endocrine disorder.^26^ A possible explanation is GERD symptoms can cause irritation of the posterior throat wall and thyroid disease.^26^ On the other hand, parietal cell antibody is an immunoglobulin that can cause damage to gastric parietal cells and functions and is closely related to GERD, atrophic gastritis, and a series of autoimmune diseases, including Hashimoto’s thyroiditis.^27^

 This study has some limitations. The first limitation is the small sample size due to insufficientbudget, and another limitation is the lack of molecular study mechanism underlying the association between *H. pylori* infectionandHashimoto’s thyroiditis, especially CagA expressing strains. Since the bacterial virulence strains are different in various countries and CagA is common in Western and East Asian countries, the detection of CagA gives useful information about the complications of *H. pylori* infection that are not diagnosed by fecal antigen tests. It suggested screening of *H. pylori* infection detection by non-invasive, even invasive tests for patients with Hashimoto’s thyroiditis who are asymptomatic for gastrointestinal diseases, especially in developing and underdeveloped countries and autoimmune thyroid patients who are on high doses of thyroxin.

## Conclusion

 In conclusion, infection with *H. pylori* is possibly one of the environmental factors responsible for the pathogenesis of Hashimoto’s thyroiditis. Therefore, these results suggest screening tests for *H. pylori* detection in patients with Hashimoto’s thyroiditis, especiallythose whoaretreatment-resistant.
